# An indigenous microalgal pool containing *Klebsormidium* sp. K39 as a stable and efficacious biotechnological strategy for *Escherichia coli* removal in urban wastewater treatment

**DOI:** 10.1002/jsfa.13918

**Published:** 2024-09-23

**Authors:** Paride Salvatore Occhipinti, Nunziatina Russo, Paola Foti, Alessandra Pino, Cinzia L Randazzo, Antonino Pollio, Cinzia Caggia

**Affiliations:** ^1^ Department of Agriculture, Food and Environment University of Catania Catania Italy; ^2^ CRIAB: Centro di Ricerca Interdipartimentale per l'implementazione dei processi di monitoraggio fisico, chimico e biologico nei sistemi di biorisanamento e di acquacoltura, Department of Medical Sciences, Surgicals and Advanced Technologies, Hygiene and Public Health ‘GF Ingrassia’, University of Catania Catania Italy; ^3^ Department of Biology University of Naples Federico II, Complesso Universitario Monte Sant'Angelo Naples Italy

**Keywords:** urban wastewater treatment, microalgae, *Escherichia coli*, phycoremediation, *Klebsormidium* sp. K39

## Abstract

**BACKGROUND:**

In recent decades the demand for freshwater has drastically increased as a consequence of population growth, economic development, climate change and pollution. Therefore, any strategy for wastewater treatment can play a role in alleviating the pressure on freshwater sources.

**RESULTS:**

In the present study an autochthonous microalgal pool (MP), isolated from a constructed wetland, was proposed as an alternative to the secondary treatment of an urban wastewater treatment system. The MP removal efficacy was compared to those obtained using *Chlorella vulgaris* and *Scenedesmus quadricauda*, against *E. coli.* Results exhibited a comparable removal efficacy and after 2 days, in samples inoculated with *E. coli* at lower density, *S. quadricauda* and *C. vulgaris* induced a decrease of 2.0 units Log and the autochthonous MP of 1.8 units Log, whereas in samples with *E. coli* at higher density the bacteria were reduced 2.8, 3.4 and 2.0 units Log by *S. quadricauda*, *C. vulgaris* and the autochthonous MP, respectively. Moreover, the identification of microalgal strains isolated from the MP revealed the presence of *Klebsormidium* sp. K39, *C. vulgaris*, *Tetradesmus obliquus* and *S. quadricauda.* Although the MP composition remained quite constant, at the end of the treatment, a different distribution among the microalgal species was observed with *Klebsormidium* sp. K39 found as dominant.

**CONCLUSION:**

The microalgal‐based wastewater treatment appears as a valuable alternative, although further investigations, based on ‘omics’ approaches, could be applied to better explore any fluctuation within the MP species composition in an *in situ* trial. © 2024 The Author(s). *Journal of the Science of Food and Agriculture* published by John Wiley & Sons Ltd on behalf of Society of Chemical Industry.

## INTRODUCTION

It has been estimated that a 40% water deficit by 2030 will occur, meaning a formidable challenge for societal and economic development.^1^ The increased need for water is a consequence of population growth, economic development, climate change and pollution,^2^ above all in the Mediterranean region, considered a vulnerable area.^3^, ^4^ Furthermore, recently Zhang and co‐workers^5^ reported that in the near future (2021–2050) global streamflow may be lower than predicted by Earth System Models, particularly in Africa, Australia and North America, taking into account also evapotranspiration effects. Worldwide, 70% of freshwater resources are destined for agricultural irrigation in arid and semi‐arid regions of the globe and in southern Europe more than 50% of total water consumption takes place in agriculture.^6^, ^7^, ^8^ The International Water Management Institute^9^ estimated that by 2025, 1.8 billion people will live in countries or regions plagued by an absolute water scarcity, which means that water availability will be lower than 100 m^3^ per inhabitant per year. In such a scenario, it appears urgent to rethink water resource management.^10^ Reclaimed water (RW) represents an extremely useful strategy in many countries.^10^, ^11^, ^12^ However, based on the quality of the treated effluent, the use of RW can cause risks for plants, soils and humans.^10^, ^13^ Scientific evidences have shown that RW can contribute to the accumulation and propagation of biological (animal and human) pathogens, phytopathogens, xenobiotic contaminants (drugs and metals) and antibiotic‐resistant genes.^14^, ^15^, ^16^, ^17^ The most referred risk for the environment is related to the increase in organic matter and salinity which, in turn, causes alterations in the structure and function of the soil microbial community.^18^, ^19^ The World Health Organization guidelines fixed safety criteria for irrigation purposes, for which RW must comply with specific standards such as physicochemical and microbiological parameters. In the EU the use of RW is under the Regulation (EU) 2020/741 on minimum requirements for water reuse, which establishes a threshold of 10 CFU (100 mL)^−1^ (<1 Log (100 mL)^−1^) of *Escherichia coli* for RW to be classified as class ‘A’, useful for irrigation of food crops.^20^

In Italy about 4000 ha are irrigated by RW,^10^ and in southern regions, such as Puglia and Sicily, several pilot‐scale projects are aimed at compensating for the lack of natural resources typical of Mediterranean areas.^21^, ^22^ Among the wastewater treatment systems, constructed wetlands (CWs) are considered environmentally sustainable, involving the use of engineered technologies designed to exploit natural processes.^12^ The CWs are effective in reducing biological oxygen demand and total suspended solids and are highly recommended as cheap secondary treatment systems, although some drawbacks still limit their spread, such as: (a) limited effect on phosphorus and nitrogen removal (especially free surface type) and (b) limited capacity to remove fecal coliform^23^ or to zero *E. coli*. Different efficacies have been reported. For instance, Green *et al*.^24^ reported a reduction up to 1000 CFU of *E. coli* per 100 mL. Diaz *et al*.^25^ found a wide variability, between 66% and 91% of *E. coli* loads retained in wetlands. An efficacious solution for a complete removal of fecal coliforms was tested by Russo and co‐workers^26^ using a UV treatment on water effluent of CW. The biotransformation of pollutants from wastewater, including xenobiotics, nutrients and CO_2_ from polluted air by macroalgae is known as phycoremediation and microalga‐based wastewater treatment is one of the most promising technologies for advanced treatment. Furthermore, as recently reported, the main advantage of using microalgae in wastewater treatment lies in their ability to produce O_2_ through photosynthesis which is essential for the growth of heterotrophic bacteria which cause the biodegradation of organic substances, reducing the energetic cost of treatment and limiting the environmental impact.^27^

The most common microalgal species used in wastewater treatment belong to the genera *Scenedesmus*, *Dunaliella*, *Phaeodactylum*, *Botryococcus*, *Oscillatoria*, *Pediastrum*, *Nitzschia*, *Cosmarium*, *Micractinium*, *Chlamydomonas* and *Actinastrum* used both as axenic culture (pure culture) or as mixed culture.^28^, ^29^ Among them, *Chlorella* sp. and *Scenedesmus* sp. are known to be highly resistant to different pollutants, such as polycyclic aromatic compounds, hydrocarbons, phenolic compounds and organic solvents,^30^ and naturally dominate most continuous microalgal‐based treatment systems, particularly in bacterial and microalgal consortia.^31^ Furthermore, *Chlorella vulgaris*, because of its good acclimatization to a wide range of environmental conditions, its immobilization and biosorption capacity, with high nutrient removal rate,^32^ is the most used species in bioremediation applications. Therefore, the concept of microalgal biorefinery is relatively new and few data are available on exploitation of microalgal pool naturally selected in a specific environment.

The aim of the study reported here was to evaluate the performance of *E. coli* removal efficacy of microalga‐based wastewater treatment based on an autochthonous microalgal pool (MP), obtained from a CW located in Sicily, as an alternative to secondary treatment. In particular, the MP removal efficacy against intentionally inoculated *E. coli* was compared to that of both *Chlorella vulgaris* and *Scenedesmus quadricauda* in single cultures in Imhoff tank autoclaved water (ITAW) sampled at the same CW system.

## MATERIALS AND METHODS

### Microbial strains, media and cultivation conditions

*Chlorella vulgaris* ACUF863, *Chlorella vulgaris* ACUF110 and *Scenedesmus quadricauda* ACUF581 strains, belonging to the Algal Collection of ‘Federico II’ Naples University (ACUF), were cultivated photo‐autotrophically in 250 mL sterile flasks. *C. vulgaris* ACUF863 and *S. quadricauda* ACUF581 (at final cell density of 3.5 × 10^4^ cells mL^−1^) were singly inoculated into 100 mL of bold basal medium (BBM) broth (2.94 mmol L^−1^ NaNO_3_, 0.17 mmol L^−1^ CaCl_2_∙2H_2_O, 0.30 mmol L^−1^ MgSO_4_∙7H_2_O, 0.43 mmol L^−1^ K_2_HPO_4_, 1.29 mmol L^−1^ KH_2_PO_4_, 0.43 mmol L^−1^ NaCl, 8.5 μmol L^−1^ EDTA, 0.9 μmol L^−1^ FeSO_4_, 9 μmol L^−1^ H_3_BO_3_, 1.50 μmol L^−1^ ZnSO_4_·7H_2_O, 0.36 μmol L^−1^ MnCl·4H_2_O, 0.26 μmol L^−1^ MoO_3_, 0.31 μmol L^−1^ CuSO_4_·5H_2_O, 0.084 μmol L^−1^ Co(NO_3_)_2_·6H_2_O) and incubated at 25 ± 2 °C under axenic conditions, with a 16:8 (day:night) photoperiod, under LED light (25.000 lx) and shaking (150 rpm). The final cell density was confirmed by Bürker chamber counting.

The MP was obtained from the free water surface (FWS) pond in a wetland plant located in a farmhouse in Sicily, through the serial dilution method. In detail, a water sample from FWS was diluted 1:1000 in sterilized BBM and incubated at the conditions reported above. After 10 days, 200 μL of diluted sample was purified by streaking on BBM agar medium, supplemented with rifampicin (50 mg L^−1^) and carbenzadin (5 μg mL^−1^). Plates were incubated for 3 weeks at 25 ± 2 °C, under a photon flux density of 100 μmol photons m^−2^ s^−1^ and with a 16:8 (day:night) photoperiod. Simultaneously, 1 mL of raw diluted (1:1000) water sample from FWS was transferred into a 24‐well flat‐bottom tissue culture plate (Falcon, Becton Dickinson and Company, Franklin Lakes, NJ, USA) in order to isolate microalgae using a micropipetting technique under an inverted microscope (Fluovert, Leitz Wetzlar Germany, type 307‐148.002). The microalgal isolates were purified by streaking on BBM agar medium, supplemented with rifampicin and carbenzadin (5 and 50 mg L^−1^, respectively) and incubated at the same conditions as reported above. To verify the axenicity, purified microalgal isolates and MP were streaked on BBM agar medium supplemented with glucose (18 mg L^−1^), according to Guillard,^33^ and incubated for 72 h at 37 °C in darkness. Finally, ten purified microalgal isolates and purified colonies from MP were singly transferred into sterile flasks containing BBM broth and incubated at the conditions reported above. To follow the microalgal dynamics of species present in the MP during the experimental trials, a visual snapshot was obtained by microscopic observations. In detail, 10 μL of undiluted water sample, before, during and after the trials was laid on a glass microscope slide and observed by an Olympus BX40 system (Olympus Italia S.r.l., Segrate Milano) at ×10, ×40 and ×100 magnification.

The *E. coli* ATCC25922 strain was used as target microorganism in wastewater samples. The culture was revitalized in brain heart infusion broth (BHI; Scharlau Microbiology, Scharlab, Spain) and incubated overnight at 37 °C to obtain a final cell density of 9 Log CFU mL^−1^, evaluated by the serial dilutions method into Chromatic™ EC X‐GLUC agar (Liofilchem, Italy).

### Wastewater sampling

Wastewater samples were obtained from the Imhoff tank of the same CW plant, located in Sicily (Italy). Samples were collected, using sterile glass bottles, and immediately transferred to the Laboratory of Microbiology at the Department of Agricultural, Food and Environment (University of Catania).

Wastewater samples were subjected to pH, electrical conductivity, total suspended solids, nitrate‐nitrogen (NO^3−^ single bond N), sulfate (SO_4_^−2^), total phosphorus, five‐day biochemical oxygen demand and salt (Na^+^, K^+^, Ca^2+^) determinations and data are reported in supporting information, Table S1. Microbiological analyses were carried out following the membrane filtration method,^34^ and for *E. coli* detection and counting, 100 mL of water sample was filtered on membrane filters (0.45 μm pores, cellulose, Merck, Germany) and poured in RAPID *E. coli* 2 agar plates (Bio‐Rad, Italy), incubated at 37 °C for 24 h.

### Experimental design

**Figure 1:**
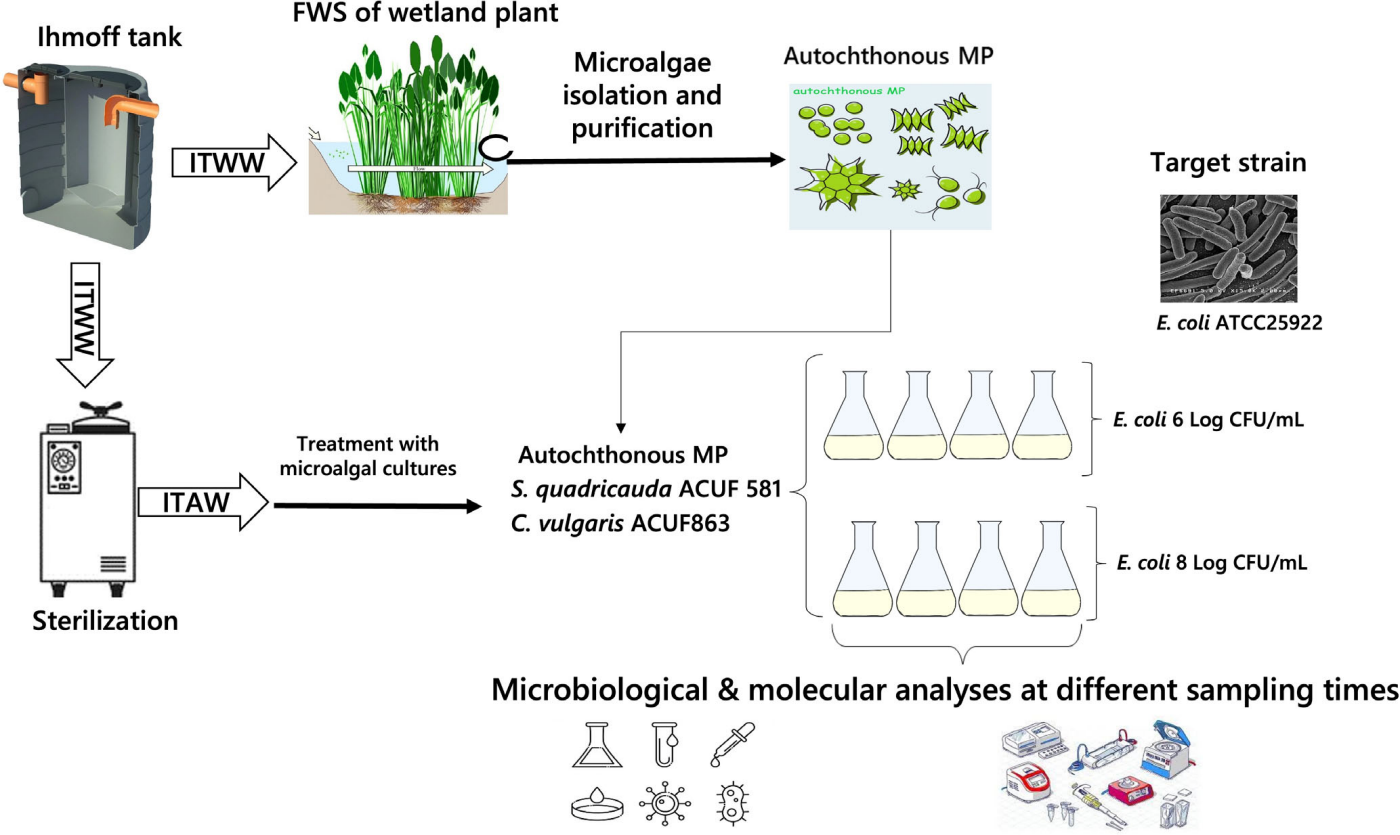
Experimental design (ITWW, Imhoff tank wastewater; ITAW, Imhoff tank autoclaved water; FWS, free water surface pond).

Water samples, obtained from the Imhoff tank, were grossly filtered through a 5–10 μm pore size Fisherbrand™ cellulose filter paper (Thermo Fisher Scientific, Waltham, MA, USA) and sterilized at 121 °C for 20 min, affording ITAW. Sterile flasks (300 mL) containing 150 mL of ITAW sample were singly inoculated with *C. vulgaris* ACUF863, *S. quadricauda* ACUF581 and the autochthonous MP, at a final concentration of 5 Log cells mL^−1^. In order to evaluate the removal efficacy of each microalgal culture, immediately after, *E. coli* ATCC25922, cultured at 37 °C for 24 h in BHI, was inoculated in each flask at final concentrations of 6 or 8 Log CFU mL^−1^ (Fig. 1). ITAW samples inoculated with single microalgal culture were used as controls whereas ITAW samples inoculated with a fresh *E. coli* ATCC25922 culture, at a final density of 6 or 8 Log CFU mL^−1^, were used as positive controls. The flasks were kept at 25 ± 2 °C, under a photon flux density of 100 μmol photons m^−2^ s^−1^ and with a 16:8 (day:night) photoperiod for 12 days. The optical density of samples, differently treated, at different sampling times, was determined using a spectrophotometer (Cary 100 Scan UV–visible, Agilent, CA, USA) at 550 nm.

Samples were taken at 0, 2, 4, 6, 8 and 12 days after inoculum and microbiological analyses were performed in triplicate.

### pH monitoring

ITAW samples, inoculated with autochthonous MP, *C. vulgaris* ACUF863 and *S. quadricauda* ACUF581, with and without *E. coli* ATCC25922, were taken at 0, 2, 4, 6, 8 and 12 days after inoculum, and subjected to pH determination. The pH value was determined at 25 °C using an Xs pH50 instrument. The analysis was performed in triplicate and results are reported as mean pH and standard deviation.

### *E. coli* removal efficacy

In order to evaluate the bacterial removal efficacy, ITAW samples inoculated with autochthonous MP, *C. vulgaris* ACUF863, *S. quadricauda* ACUF581 and immediately after with *E. coli* ATCC25922 were taken at 0, 2, 4, 6, 8 and 12 days. Analysis was performed according to APHA guidelines,^34^ and *E. coli* was enumerated according to the ISO 9308‐1 procedure,^35^ using Chromatic™ EC X‐GLUC agar, incubated at 37 ± 2 °C for 48 h. Analysis was performed in triplicate and results expressed as mean log_10_ CFU per unit of volume and standard deviation.

### Identification of strains isolated from autochthonous MP

Based on phenotypical and microscopic traits, two microalgal isolates (M1 and M2) were selected and subjected to total DNA extraction, following the CTAB method.^36^ DNA was amplified using the primer pairs AV‐rbcL_RH1‐f (ATGTCACCACAAACAGAAACTAAAGC) and AV‐rbcL_1385r (AATTCAAATTTAATTTCTTTCC), targeting the *rbcL* gene for green algae,^37^ and primers V0‐V1_63f (CAG GCC TAA CAC ATG CAA GTC) and V6‐1073r (ACGAGCTGACGACARCCATG), targeting the 16S rRNA gene for cyanobacteria.^38^, ^39^ PCR was performed in a final volume of 50 μL, containing 30 ng of DNA template, 2.5 U of Taq DNA polymerase (Invitrogen, Italy), 10 mmol L^−1^ Tris–HCl (pH 8.4), 50 mmol L^−1^ KCl, 1.5 mmol L^−1^ MgCl_2_, 200 μmol L^−1^ of each dNTPs and 100 mmol L^−1^ of each primer. Amplification reactions were performed using a T100 thermal cycler (Bio‐Rad, Hercules, CA, USA) as follows: 1 cycle at 94 °C for 4 min; 35 cycles at 94 °C for 60 s, 45 °C for 2 min, 65 °C for 3 min, 1 cycle at 72 °C for 5 min (for AV‐rbcL_RH1‐f and AV‐rbcL_1385r primer pairs); 1 cycle at 95 °C for 1 min; 30 cycles at 95 °C for 60 s, 60.5 °C for 1 min, 72 °C for 1.5 min, 1 cycle at 72 °C for 5 min for V0‐V1_63f (CAG GCC TAA CAC ATG CAA GTC) and V6‐1073r (ACGAGCTGACGACARCCATG) primer pairs. Amplicons were analyzed by electrophoresis in 1.0% (w/v) agarose gel in TBE 1× buffer (89 mmol L^−1^ Tris–borate, 89 mmol L^−1^ boric acid, 2 mmol L^−1^ EDTA; pH 8.0), running at 100 V for 45 min, and visualized after staining with Gel Red Nucleic Acid Stain (Biotium, Inc., Fremont, CA, USA).

PCR products, obtained by primer pairs targeting the *rbcL* gene, were purified using a Qiaquick PCR purification kit (Qiagen Hilden, Germany), and subjected to sequencing, performed by an external service (Eurofins Genomics, Vimodrone, Italy). Taxonomic identification was assessed by sequence analysis of the *rbcL* gene using the Basic Local Alignment Search Tool (BLASTn) software in the standard databases (nucleotide collection:nr/nt).

### PCR‐DGGE analysis

Microalgal isolates, obtained from the autochthonous MP and from the ITAW samples inoculated with the autochthonous MP plus *E. coli* at 8 Log CFU mL^−1^, were subjected to PCR denaturing gradient gel electrophoresis (DGGE) analysis. Total DNA was extracted following the CTAB DNA method.^36^ PCR products were obtained using the primer pairs Euk1A (CTGGTTGATCCTGCCAG) and Euk516r‐GC (ACCAGACTTGCCCTCCCGCCCGGGGCGCGCCCCGGGCGGGGCGGGGGCACGGGGGG), amplifying a 560 bp fragment of the eukaryotic 18S,^40^ and the primer pairs GC‐16S353F (CGCCCGCCGCGCGCGGCGGGCGGGGCGGGGGCACGGGGGGAGCAGTGGGGAATTTTCCGC‐) and CYA781RA (GACTACTGGGGT ATCTAATCCCATT), amplifying a 409 bp fragment of the cyanobacterial 16S ribosomal DNA,^41^, ^42^ and PCR reactions were performed as previously reported.^40^, ^41^

DGGE analysis of PCR amplicons was performed following the protocol described by Dìez and co‐workers^40^ and by Granada‐Moreno *et al*.,^43^ using the DCode System (Bio‐Rad Laboratories, Hercules, CA). The polyacrylamide gel consisted of 8% (w/v) polyacrylamide (37.5:1 acrylamide–bisacrylamide) in 0.5× TAE buffer. Denaturing acrylamide of 100% was defined as 7 mol L^−1^ urea and 40% (v/v) formamide. The gels were poured from the top using a gradient maker and the pump (Econopump, Bio‐Rad) was set at a rate of 4.5 mL min^−1^. The gradient was set at 40–65% for the amplicons generated by the Euk1A/Euk516r‐GC primers and at 30–40% for the amplicons generated by GC‐16S353F/CYA781RA primers. Electrophoresis was performed for 16 h at a voltage of 90–100 V in a 0.5× TAE buffer at a constant temperature (60 °C). Gels were stained with silver nitrate, according to Sanguinetti and Simpson^44^ and Randazzo and co‐workers.^45^

### Statistical analysis

One‐way analysis of variance (ANOVA) and Tukey's HSD *post hoc* test for means separation were performed using Statistica ETL software (version 10, StatSoft. inc., Tulsa, OK, USA). The significance level was set at *P* ≤ 0.05.

## RESULTS

### pH values of ITAW samples differently inoculated with *E. coli* ATCC25922 and treated with different microalgal cultures

**Figure 2:**
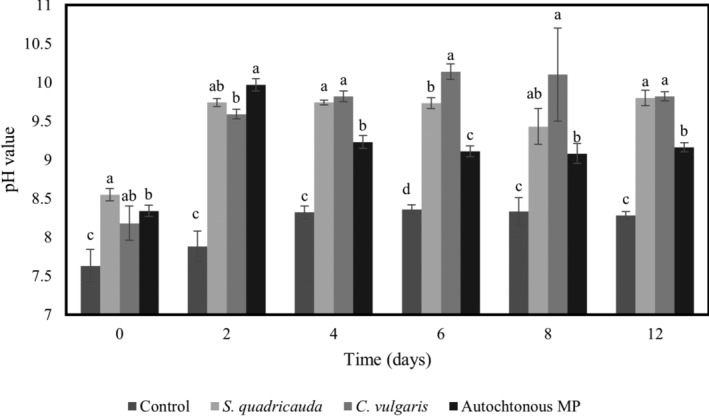
pH values determined in ITAW samples inoculated with *E. coli* ATCC25922 at 6 Log CFU mL^−1^ treated with different microalgal cultures (*C. vulgaris* ACUF863, *S. quadricauda* ACUF581 and autochthonous MP) at 0, 2, 4, 6, 8 and 12 days. Data are expressed as means of three replicates ± SD. Values of the same time followed by different letters are significantly different (*P* ≤ 0.05 in one‐way ANOVA).

The pH values determined in ITAW samples, inoculated with *E. coli* ATCC25922 (6 Log CFU mL^−1^) and with different microalgae (*C. vulgaris* ACUF863, *S. quadricauda* ACUF581 or autochthonous MP), after 0, 2, 4, 6, 8 and 12 days, are reported in Fig. 2. Overall, at each sampling time, ITAW samples inoculated with microalgae exhibited pH values 1.5 points higher than those observed in ITAW samples exclusively inoculated with the *E. coli* ATCC25922 strain. Starting from day 4, the ITAW samples inoculated with the autochthonous MP showed the lowest pH values, except at day 8 when the pH was found to be similar to that for ITAW sample inoculated with *S. quadricauda* ACUF581.

**Figure 3:**
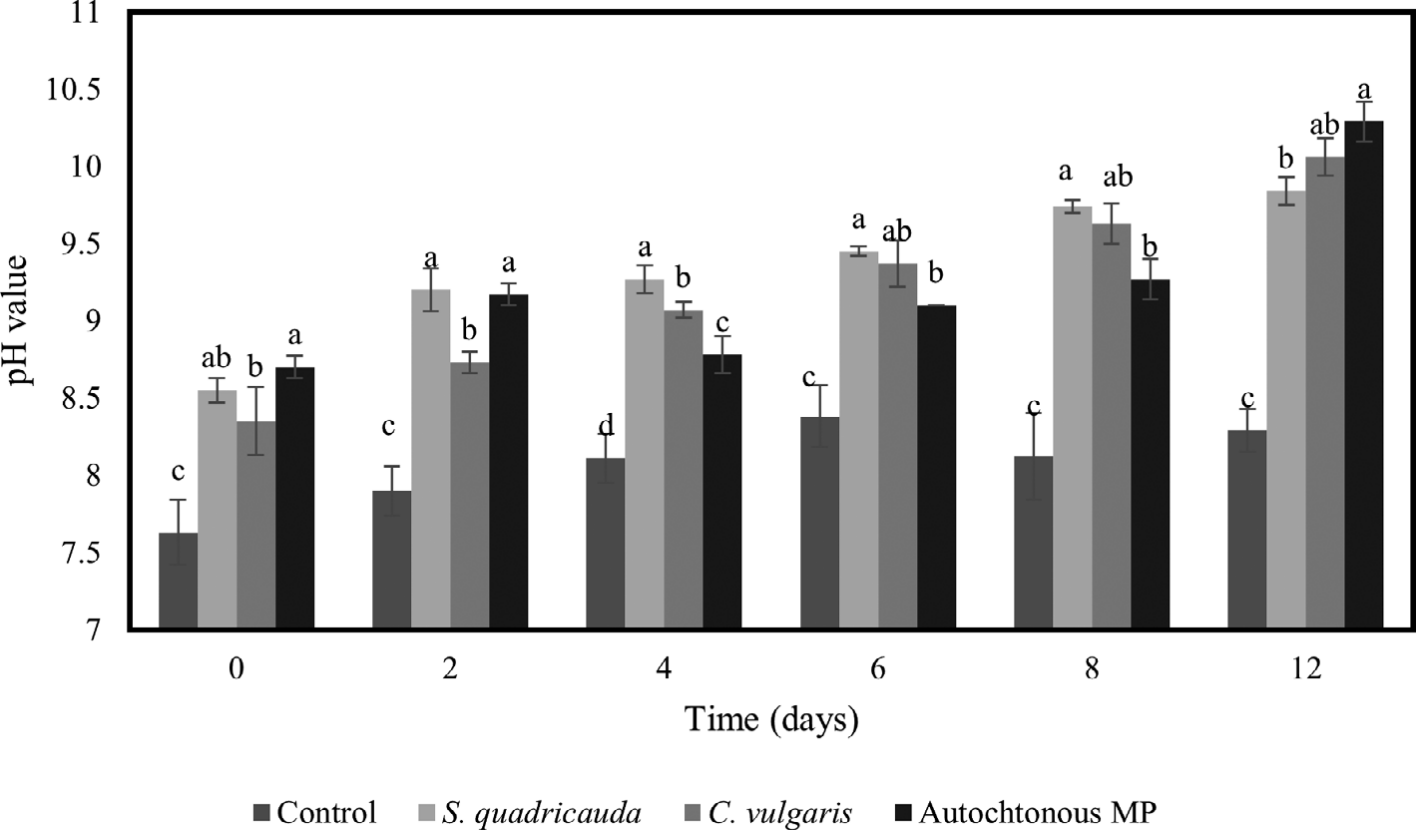
pH values determined in ITAW samples inoculated with *E. coli* ATCC25922 at 8 Log CFU mL^−1^ treated with different microalgal strains (*C. vulgaris* ACUF863, *S. quadricauda* ACUF581 and autochthonous MP) at 0, 2, 4, 6, 8 and 12 days. Data are expressed as means of three replicates ± SD. Values of the same time followed by different letters are significantly different (*P* ≤ 0.05 in one‐way ANOVA).

The pH values determined in ITAW samples inoculated with target bacteria at higher density, 8 Log CFU mL^−1^, and differently treated with the tested microalgal cultures at 0, 2, 4, 6, 8 and 12 days after inoculum are reported in Fig. 3. Overall, the mean pH values of samples inoculated with microalgae were significantly higher (of about 1.5 points) than those of controls (samples inoculated exclusively with *E. coli* ATCC25922), at each sampling time. Focusing on day 6, samples showed pH values of 9.37, 9.45, 9.10 and 8.38, respectively, for *C. vulgaris* ACUF863, *S. quadricauda* ACUF581, MP and control sample (inoculated exclusively with *E. coli*). Moreover, ITAW samples inoculated with autochthonous MP showed pH values similar to those of samples inoculated with *C. vulgaris* ACUF863 (at 4, 6, 8 and 12 days after inoculum) and to those of samples inoculated with *S. quadricauda* ACUF58 (at 2, 6 and 8 days after inoculum).

### Microalgal concentration in ITAW samples at 0, 2, 6, 8 and 12 days from inoculum

The microalgal counts, detected in ITAW samples inoculated with *C. vulgaris* ACUF863, *S. quadricauda* ACUF581 and the autochthonous MP, were determined by Bürker chamber counting cell (supporting information, Table S2). Overall, no significant difference in microalgal concentration was observed between the MP‐ and *C. vulgaris* ACUF863‐inoculated samples, at any sampling time, showing a mean value of 6.00 Log cells mL^−1^, whereas ITAW samples treated with *S. quadricauda* ACUF581 showed lower mean values (5.63 Log cells mL^−1^).

### Microalgal densities detected in ITAW samples inoculated with *E. coli* ATCC25922 at different concentrations

In ITAW samples inoculated with *E. coli* ATCC25922 (at 6 or 8 Log CFU mL^−1^) and with the different microalgal cultures (*C. vulgaris* ACUF863, *S. quadricauda* ACUF581 or autochthonous MP), the microalgal counts were performed, by Bürker chamber counting cell, after 0, 8 and 12 days from the inoculum (supporting information, Fig. S1). Even though high variability was observed among the ITAW samples, a good growth performance was exhibited by autochthonous MP in the presence of *E. coli* ATCC25922 at both 6 and 8 Log CFU mL^−1^. In detail, after 8 days from inoculum, no significant difference was detected among MP, *C. vulgaris* ACUF863 and *S. quadricauda* ACUF581 counts in ITAW samples inoculated with *E. coli* at both densities. At that sampling time, it is interesting to highlight that the microalgal counts in samples inoculated with autochthonous MP reached mean values higher than 6.0 Log cells mL^−1^, when *E. coli* ATCC25922 was inoculated at both 6 and 8 Log CFU mL^−1^. At the lower *E. coli* ATCC25922 tested concentration, ITAW samples inoculated with *S. quadricauda* ACUF581 showed the lowest microalgal count (5.34 Log cells mL^−1^), lower than 0.73 Log cells mL^−1^ compared to MP. Similar results were found after 12 days, when in ITAW sample inoculated with *S. quadricauda* ACUF581, microalgal densities were found to be lower than those for MP at both *E. coli* ATCC25922 tested densities. Overall, the microalgal densities in samples inoculated with MP were always similar to those in samples inoculated with *C. vulgaris* ACUF863.

### *E. coli* removal efficacy by microalgal cultures

**Figure 4:**
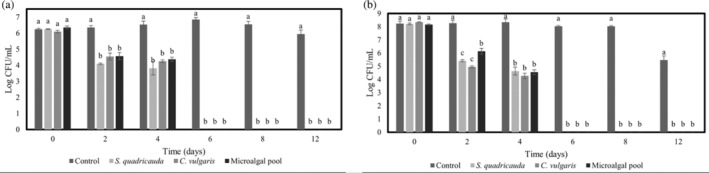
*E. coli* density determined in ITAW samples inoculated with *E. coli* ATCC25922 at 6 Log CFU mL^−1^ (a) and 8 Log CFU mL^−1^ (b) treated with different microalgal strains (*C. vulgaris* ACUF863, *S. quadricauda* ACUF581 or autochthonous MP) at initial time and after 2, 4, 6, 8 and 12 days from inoculation. Data are expressed as means ± SD. Mean values with different letters at the same sampling time are statistically different (*P* ≤ 0.05).

The cell density of *E. coli* ATCC25922 determined in ITAW samples, in uninoculated (control) and in samples inoculated with different microalgal cultures (*C. vulgaris* ACUF863, *S. quadricauda* ACUF581 or autochthonous MP) after 0, 2, 4, 6, 8 and 12 days, are shown in Fig. 4. Overall, a significant reduction of *E. coli* ATCC25922 cell density was observed in all tested samples, with the exception of controls. In detail, in samples inoculated with *E. coli* ATCC25922 at lower density (6 Log CFU mL^−1^), no significant difference was detected in the removal efficacy of the tested microalgae (Fig. 4(a)). In detail, 2 days after the microalgal inoculum, *S. quadricauda* ACUF581 and *C. vulgaris* ACUF863 induced a decrease of 2.07 units Log of *E. coli* ATCC25922 viable cells, whereas autochthonous MP induced a decrease of 1.85 units Log. In samples of the same trial, *E. coli* ATCC25922 viable cells were found to be below the detection limit starting from day 6. A different trend was observed in uninoculated sample (control), where the *E. coli* density was found to be almost constant until day 8, to reach, after 12 days, a value of 5.94 Log CFU mL^−1^. In ITAW samples inoculated with *E. coli* ATCC25922 at higher density (8 Log CFU mL^−1^), each microalgal culture exhibited similar removal efficacy with a significant reduction of the target bacteria (Fig. 4(b)). In detail, after 2 days, in samples treated with *S. quadricauda* ACUF581, *C. vulgaris* ACUF863 and autochthonous MP the target bacteria were reduced by 2.8, 3.4 and 2.0 units Log, respectively. After 6 days, *E. coli* was never detected in any microalga‐treated ITAW samples, while its density was found at a mean value of 7.17 Log CFU mL^−1^ in untreated samples.

### Sequencing data and BLAST alignment

The sequencing results of M1 and M2 isolates were compared with the sequence databases by BLAST. The M1 strain (accession number OQ363409) corresponded, at 100%, to *Klebsormidium* sp. K39, whereas M2 (accession number OQ363408) corresponded at 99.6% to *Tetradesmus obliquus*.

### MP community dynamic during trials

**Figure 5:**
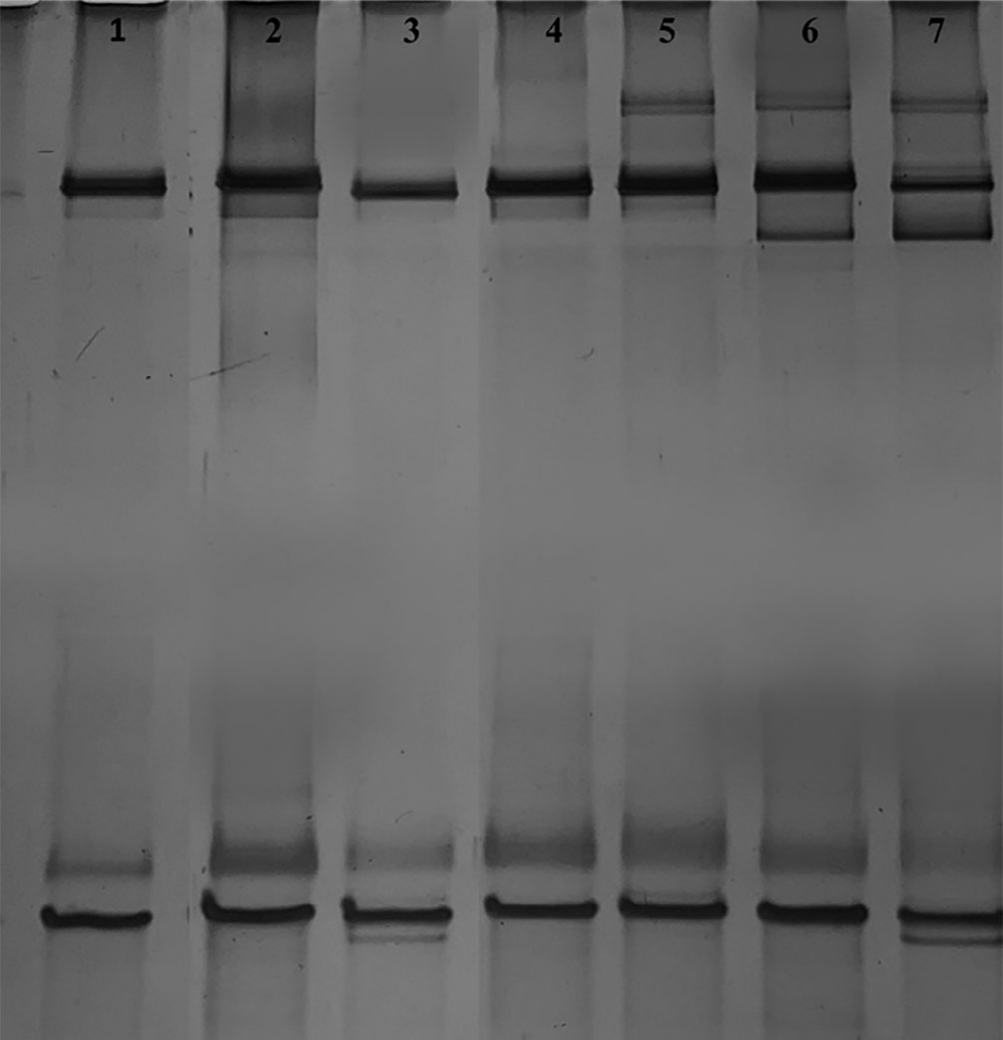
DGGE patterns of microalgal 18S rRNA gene fragments amplified using 18S primers set A (Euk1A and Euk516r‐GC). Line 1: M1 strain, identified as *Klebsormidium* sp. K39; line 2: *C. vulgaris* ACUF110 strain; line 3: M2 strain identified as *Tetradesmus obliquus*: line 4: *C. vulgaris* ACUF863: line 5: *S. quadricauda* ACUF581; line 6: ITAW samples inoculated with the higher concentration of *E. coli* ATCC25922 treated with autochthonous MP after 12 days; line 7: autochthonous MP cultured on BBM.

In order to highlight the dynamic of the microalgal community, the MP growth in the medium, the MP inoculated in ITAW sample added with *E. coli* at 8 Log CFU mL^−1^ and the microalgal isolates were subjected to PCR‐DGGE analysis and the obtained profiles were compared. No amplification was obtained using cyanobacteria 16S ribosomal DNA (rDNA)‐specific set B primers (GC‐16S353F and CYA781RA). Regarding the eukaryotic community profiles, obtained by primers 18S rRNA gene amplicon pairs Euk1A and Euk51rev, although the PCR amplification yielded a single band, the DGGE analysis revealed the presence of distinct bands (Fig. 5).

In detail, as shown in Fig. 5, the DGGE profiles confirmed the presence of species belonging to *Klebsormidium* sp. K39 and to *T. obliquus* rather than *C. vulgaris* and *S. quadricauda*.

**Figure 6:**
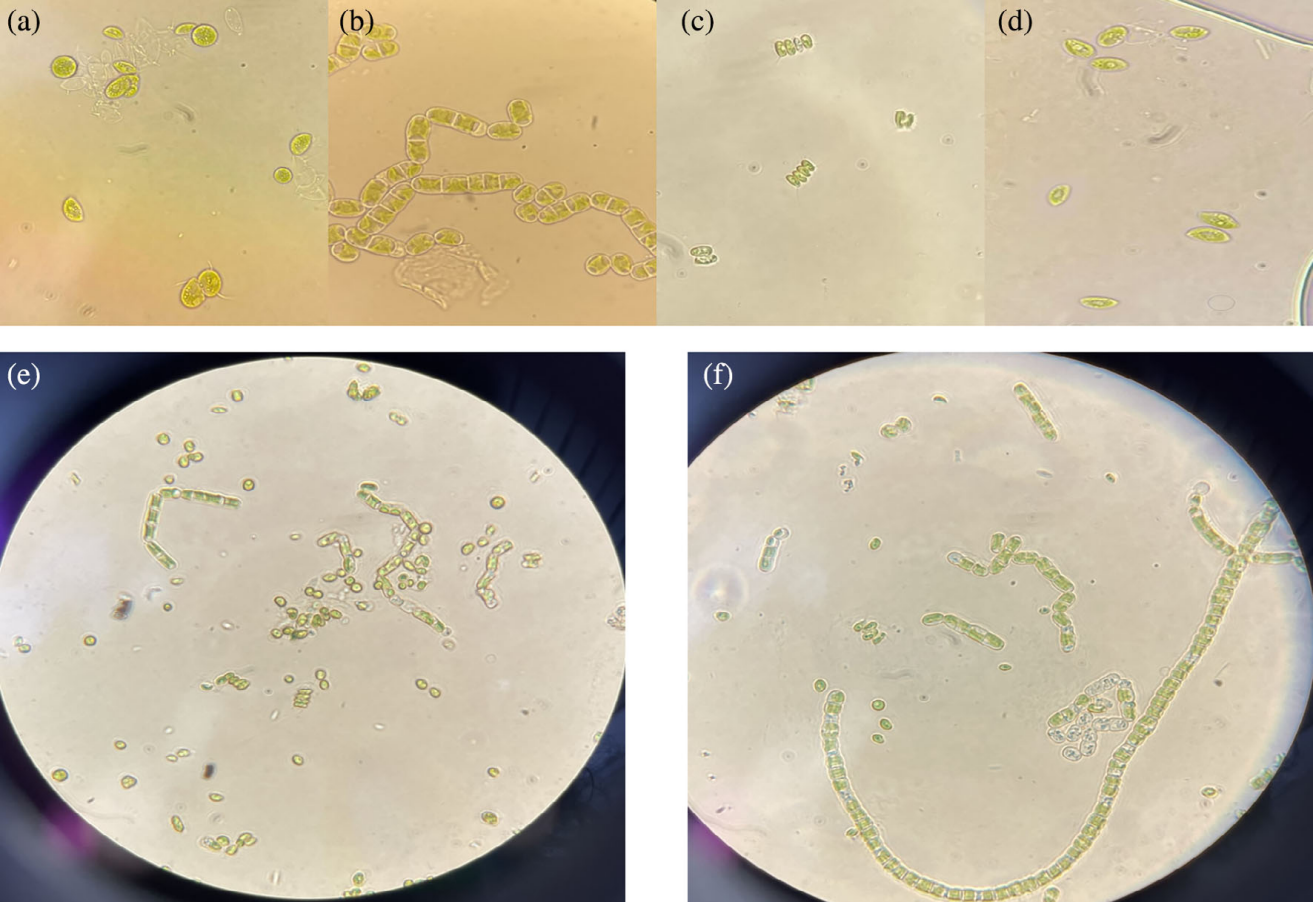
Microscope images of microalgal strains and autochthonous MP used in the present study. (a) *C. vulgaris* at ×100 magnification; (b) *Klebsormidium* sp. K39 at ×100 magnification; (c) *S. quadricauda* at ×100 magnification; (d) *T. obliquus* at ×100 magnification; (e) autochthonous MP cultured on BBM visualized at ×40 magnification; (f) ITAW samples inoculated with the higher concentration of *E. coli* ATCC25922 treated with autochthonous MP after 12 days visualized at ×40 magnification.

Furthermore, comparing profiles obtained from ITAW samples inoculated with the higher concentration of *E. coli* ATCC25922 treated with autochthonous MP after 12 days (line 6, Fig. 5) with those obtained by autochthonous MP cultured on BBM (line 7, Fig. 5), it is interesting to underline the disappearance of the lightest band, corresponding to the *T. obliquus* profile (line 3, Fig. 5). These results are in accordance with microscope images obtained from fresh autochthonous MP, cultured in BBM (Fig. 6(e)), and with those obtained from ITAW samples inoculated with the higher concentration of *E. coli* ATCC25922 after 12 days of treatment with the autochthonous MP (Fig. 6(f)). In particular, as shown in Fig. 4, when the autochthonous MP was cultured on BBM, a quite uniform distribution of each microalgal species was observed, even though *Chlorella* sp. was found as prevalent, whilst in ITAW samples inoculated with *E. coli*, after 12 days of treatment with autochthonous MP, a different species distribution was observed, with *Klebsormidium* sp. K39 found as dominant microalga in place of *Chlorella* sp. and with reduction and disappearance of *S. quadricauda* and *T. obliquus*, respectively.

## DISCUSSION

Because of the metabolic flexibility of microalgae, i.e. their ability to perform photoautotrophic, mixotrophic or heterotrophic metabolism, they represent a promising biological system for treating a variety of sources of wastewater.^46^, ^47^, ^48^ Moreover the microalgal photosynthetic activity contributes to the inhibition of bacterial growth by increasing pH, temperature and dissolved oxygen concentration.^31^ In this study, a wastewater treatment based on an autochthonous MP, isolated from the FWS pond of a CW plant, was compared to treatments performed using single *C. vulgaris* ACUF863 or *S. quadricauda* ACUF581 in sterilized wastewater samples taken from the same CW plant. In particular, the *E. coli* removal efficacy was tested in a 12‐day period starting from two initial bacterial densities to evaluate the removal effect in specific physicochemical conditions. Results related to microalgal densities reveal that in ITAW samples without *E. coli*, the MP reached similar cell densities to those detected for *C. vulgaris* ACUF863, and higher than those detected for *S. quadricauda* ACUF581 after 2, 6 and 12 days. These findings highlighted that autochthonous MP is well adapted to specific conditions and confirmed the great ability of the two species, namely *C. vulgaris* and *S. quadricauda*, to easily acclimate, as already reported.^28^, ^29^, ^32^ Nevertheless, compared to monocultures, microalgal polycultures can resist invasive species and represent a more robust system able to adapt to environmental fluctuations.^49^ In the present study, according to previous findings,^50^, ^51^, ^52^ the microalgal densities in samples inoculated with MP exhibited values quite similar to those in samples inoculated with *C. vulgaris* plus *E. coli* at both concentrations, while higher microalgal concentrations were found compared with those in control samples inoculated with *S. quadricauda*, or with *S. quadricauda* plus *E. coli* at both concentrations. Focusing on *E. coli* removal efficacy, in the ITAW samples inoculated with *E. coli* ATCC25922 at both densities, treated with each microalgal culture, the *E. coli* was not detected starting from day 6. Concurrently, the pH values in samples treated with microalgae were about 2 points higher than in control samples inoculated only with *E. coli*. Indeed, one of main mechanism involved in *E. coli* removal is related to the increase of pH, mainly due to the microalgal photosynthetic activity.^31^, ^53^, ^54^ For example, Schumacher *et al*.^47^ found a decrease of total coliforms and *E. coli* of four and six orders of magnitude when pH increased from 8.4 to 10.5, respectively. The key role of alkaline pH in *E. coli* removal was also confirmed by Heubeck *et al*.,^48^ who observed significantly higher *E. coli* removals at pH 9.5 (~100%) than at pH 8 (~50%) in a high‐rate algal pond treating domestic wastewater. Instead, Posadas *et al*. and Zitnik *et al*. reported that when the pH of the medium was adjusted to 7–8, a mutualistic relationship between microalgae and *E. coli* is observed, without any bacterial removal effect.^55^, ^56^ Overall, as extensively observed, any physicochemical parameter favorable for algal growth is generally unfavorable for virus, amoeba, protozoa or bacterial survival.^57^ Focusing on ITAW samples inoculated with *E. coli* at lower density, all treatments exhibited a similar removal efficacy. In each treatment, *E. coli* was not detected starting from day 6 after inoculum. A similar trend was observed in ITAW samples inoculated with *E. coli* at higher density, at any sampling points, except in samples treated with autochthonous MP, which registered an *E. coli* density of 6.13 Log CFU mL^−1^, 1.18 and 0.72 units higher than those determined in samples treated with *C. vulgaris* and *S. quadricauda*, respectively. These data confirmed that the removal efficacy of the autochthonous MP is comparable to that of most common species largely used in microalga‐based wastewater treatments, also in different stress conditions, according to Colak and Kaya, who reported a coliform removal rate of 99% in high‐rate algal ponds, and in agreement with Abdel‐Raouf and co‐workers, who reported, in stabilization ponds, a removal of coliforms of up to 99.6%.^58^, ^59^

Focusing on PCR‐DGGE analyses, results confirmed quite a stability of the autochthonous MP until the end of the experimental trial. In particular, only the 18S rDNA gene was amplified and even if for each single strain only one amplicon was obtained, the DGGE revealed multiple distinct bands, according to Lakaniemi *et al*.^60^, ^61^ In detail, results showed the presence of *Klebsormidium* and *Tetradesmus* genera, the latter genus largely exploited in synthetic or municipal wastewaters.^62^ Furthermore, *Klebsormidium* sp. K39 resulted as the main microalgal species present in the autochthonous MP. The observed stability of MP during previous preliminary observations confirms that mixed culture would have a better resilience to variable environmental conditions. Results of the present study are in accordance with those recently reported by Li *et al*., who found that the co‐culture of *Klebsormidium* sp. and *Spirogyra* sp., two filamentous microalgal species with different cell sizes, represents a valuable model of filamentous combination to treat tertiary effluent.^63^

The cell density of *Klebsormidium* sp. appeared to gradually increase during the trials, in accordance with the microscope observations and with results of Liu *et al*. who found that filamentous algae exhibit advantages in wastewater treatment over unicellular microalgae for their higher resistance to predation rather than easier harvesting.^64^ Different behaviors were observed for *T. obliquus*, the most common genus of green microalgae in freshwater environments, that disappeared in ITAW samples treated with autochthonous MP after 12 days.

Overall, considering the efficacy of the treatment, within the concept of the microalgal refinery approach, it is important to perform a cost–benefit analysis of the whole process, taking into account the engineering costs and the source of income resulting from microalgal biomass production.^65^

## CONCLUSION

In this study a suitable solution for a wastewater treatment based on an autochthonous MP was compared to treatments based on *C. vulgaris* and *S. quadricauda*. The autochthonous MP was characterized as mainly composed of four species belonging to green algae (Chlorophyceae), namely *Klebsormidium* spp., *Chlorella* spp., *Tetradesmus* spp. and *Scenedesmus* spp., and highlighted interesting *E. coli* removal efficiency, lowering the bacterial density to values compliant with EU regulation limits. Furthermore, the composition of autochthonous MP remained quite constant, although a slight variation, as species ratio, between initial and final samplings was observed, highlighting *Klebsormidium* as the main species at the end of the trial. Therefore, further investigations, based on ‘omics’ approaches (such as genomics, transcriptomics, proteomics, metabolomics), could be applied to produce valuable data to better explore any fluctuation within the MP species composition.

## FUNDING INFORMATION

This study was supported by the European Union (NextGeneration EU), through the MUR‐PNRR project ‘Sustainable management of natural resources in agriculture’: SAMOTHRACE (ECS00000022) and partially by PON ‘RICERCA E INNOVAZIONE’ 2014–2020, Azione II – Obiettivo Specifico 1b – Progetto ‘Miglioramento delle produzioni agroalimentari mediterranee in condizioni di carenza di ri‐sorse idriche’ – WATER4AGRIFOOD.

## CONFLICT OF INTEREST

The authors declare that they have no known competing financial interests or personal relationships that could have appeared to influence the work reported in this paper.

## AUTHOR CONTRIBUTIONS

Conceptualization, CC, AP and CLR; methodology, APn, NR and PF; formal analysis, PSO and PF; investigation, PSO and NR; resources, CC; data curation, PSO, APn and CC; writing – original draft preparation, PSO; writing – review and editing, CC, NR and CLR; visualization, PSO and CC; supervision, CC and AP; project administration, CC, CLR; funding acquisition, CC. All authors have read and agreed to the published version of the manuscript.

## Supporting information


**Figure S1.** Microalgal counts detected in ITAW samples inoculated with *E. coli* ATCC25922, at different cell densities, and with different microalgal cultures (*C. vulgaris* ACUF863, *S. quadricauda* ACUF581 or autochthonous MP) at initial time and after 8 and 12 days from inocula. The values are means of data and three replicates. Values of the same time followed by different lowercase letters are significantly different. Values of the same treatment followed by capital letters are significantly different (*P* ≤ 0.05).

**Table S1.** Physico‐chemical and microbiological traits of Imhoff tank water samples used in the present study.
**Table S2.** Growth (as Log cells/mL) of microalgae strains (*C. vulgaris* ACUF863, *S. quadricauda* ACUF581 or autochthonous MP) in ITAW samples at initial time and after 2, 6, 8 and 12 days from inocula. Data are expressed as means of three replicates ± SD. Values at the same time followed by different letters are significantly different (*P* ≤ 0.05).

## Data Availability

The data that support the findings of this study are available from the corresponding author upon reasonable request.
