# Supplementary figures and images for: An indigenous microalgal pool containing Klebsormidium sp. K39 as a stable and efficacious biotechnological strategy for Escherichia coli removal in urban wastewater treatment
Source: J Sci Food Agric. 2024 Sep 23;105(2):1288–97. doi: 10.1002/jsfa.13918 (PMC11632170; doi:10.1002/jsfa.13918)

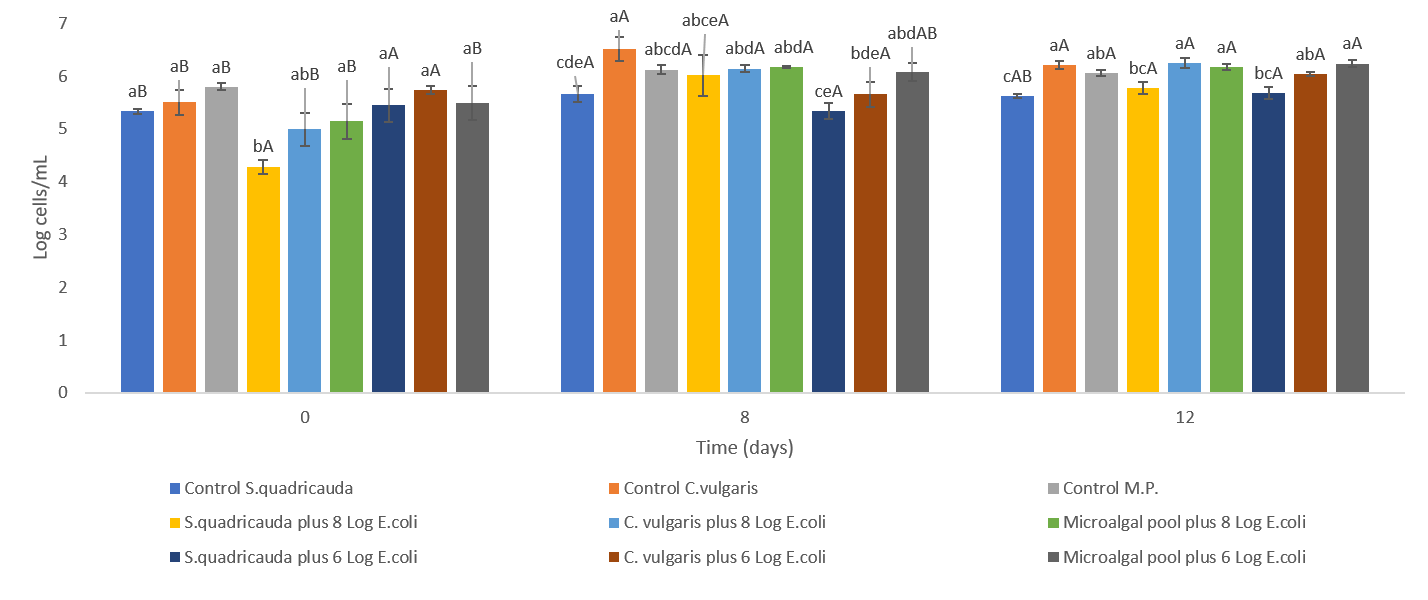

Supplement: Supplementary file 1 — Figure S1. Microalgal counts detected in ITAW samples inoculated with E. coli ATCC25922, at different cell densities, and with different microalgal cultures (C. vulgaris ACUF863, S. quadricauda ACUF581 or autochthonous MP) at initial time and after 8 and 12 days from inocula. The values are means of data and three replicates. Values of the same time followed by different lowercase letters are significantly different. Values of the same treatment followed by capital letters are significantly different (P ≤ 0.05). [file JSFA-105-1288-s001.tif]
