# Supplementary material for: An indigenous microalgal pool containing Klebsormidium sp. K39 as a stable and efficacious biotechnological strategy for Escherichia coli removal in urban wastewater treatment
Source: J Sci Food Agric. 2024 Sep 23;105(2):1288–97. doi: 10.1002/jsfa.13918 (PMC11632170; doi:10.1002/jsfa.13918)
Supplement: Supplementary file 2 — Table S1. Physico‐chemical and microbiological traits of Imhoff tank water samples used in the present study. Table S2. Growth (as Log cells/mL) of microalgae strains (C. vulgaris ACUF863, S. quadricauda ACUF581 or autochthonous MP) in ITAW samples at initial time and after 2, 6, 8 and 12 days from inocula. Data are expressed as means of three replicates ± SD. Values at the same time followed by different letters are significantly different (P ≤ 0.05). [file JSFA-105-1288-s002.docx]

**Supplementary Tables**:

**Table 1S**. Physico-chemical and microbiological traits of imhoff tank water samples used in the present study

| Parameters | Measure unit | Values |
| --- | --- | --- |
| EC | μS/cm | 3441 |
| pH | - | 6.99 |
| TSS | mg/L | 85 |
| COD | mg/L | 723 |
| N-NH_3_ | mg/L | 42.1 |
| N-NO_2_ | mg/L | 0.079 |
| N-NO_3_ | mg/L | 0.82 |
| P-PO_4_ | mg/L | 4.36 |
| *E. coli* | Log CFU/100 mL | 7.17 |
| Fluorides | mg/L | 0.747 |
| Chlorides | mg/L | 414.6 |
| Sulphates | mg/L | 376.5 |
| Sodium | mg/L | 762.4 |
| Potassium | mg/L | 24.8 |
| Magnesium | mg/L | 24.5 |
| Calcium | mg/L | 59.9 |

**Table 2S**. Growth (as Log cells/mL) of microalgae strains (*C. vulgaris* ACUF863, *S. quadricauda* ACUF581 or autochthonous MP) in ITAW samples at initial time and after 2, 6, 8 and 12 days from inocula. Data are expressed as means of three replicates ± SD. Values at the same time followed by different letters are significantly different (*p* < 0.05).

| Treatment | 0 | 2 | 6 | 8 | 12 |
| --- | --- | --- | --- | --- | --- |
| *S. quadricauda* | 5.33±0.05^b^ | 5.44±0.03^b^ | 5.80±0.06^b^ | 5.66±0.15^b^ | 5.62±0.04^b^ |
| *C. vulgaris* | 5.50±0.23^ab^ | 5.62±0.08^a^ | 6.06±0.10^a^ | 6.15±0.23^a^ | 6.21±0.07^a^ |
| Autochthonous MP | 5.80±0.07^a^ | 5.78±0.04^a^ | 6.02±0.02^a^ | 6.12±0.08^ab^ | 6.05±0.05^a^ |
